# Improving seasonal influenza vaccination for older adults

**DOI:** 10.1186/s12979-021-00224-1

**Published:** 2021-03-12

**Authors:** Graham Pawelec, Janet McElhaney

**Affiliations:** 1 Department of Immunology, University of Tübingen, Tübingen, Germany; 2 Health Sciences North Research Institute, Sudbury, Ontario Canada

Unfortunately, the worldwide COVID-19 pandemic continues unabated, but one of the few benefits conferred by the current situation is that the northern hemisphere 2020-2021 influenza season is milder and shorter than usual, and this may well  be the case for the 2021-2022  season too. This is very likely due to enforced social distancing, but may also be the result of greater uptake of influenza vaccination. The 2019–2020 data indicate that for countries where COVID-19 was best contained, the impact of influenza on public health systems and fatalities was clearly lower than would have been expected otherwise [1]. However, seasonal influenza has not gone away, and it will remain a dangerous pathogen for the foreseeable future, especially in older adults who are the most susceptible segment of the population to the serious clinical consequences of influenza disease. This is despite the availability of vaccines – but for many reasons, these are not as effective as would be desired for reliable protection, unlike the apparent situation with SARS-CoV-2 vaccines [2]. Hence there is a strong argument to increase the investment of resources for understanding the hurdles to protective influenza vaccination of older adults, and there will still be an urgent need to improve vaccines in order to prevent the 500,000 or more influenza deaths every year that occurred prior to the COVID-19 pandemic.

To this end, an important paper was recently published by the HKU-Pasteur Research Pole, University of Hong Kong, together with the WHO Collaborating Centre for Infectious Disease Epidemiology in Hong Kong, and the Centers for Disease Control and Prevention, Atlanta, GA, specifically regarding the immunogenicity of a standard high-dose of adjuvanted seasonal influenza vaccine compared with a recombinant-HA vaccine in older adults [3]. This paper describes a randomized controlled trial in which immunological monitoring of older adults was not limited to humoural responses but also included elements of cell-mediated immunity (CMI) which is essential for controlling infection, especially in older adults. Thus, cellular and antibody responses of standard-dose seasonal inactivated influenza vaccines (S-IIV) was compared with “enhanced” vaccines [MF59-adjuvanted (A-eIIV), high-dose (H-eIIV), and recombinant-hemagglutinin (HA) (R-eIIV) vaccines]. It was found that similar levels of haemagglutinin-specific IgG were induced by all the vaccines, along with increased antibody-dependent cellular cytotoxicity (ADCC). The latter was best induced by H-eIIV, whereas only A-eIIV increased HA-IgG avidity, HA-stalk IgG and ADCC activity. Importantly, polyfunctional CD4+ and CD8+ T cell responses were induced by all enhanced vaccines, but not by S-IIV. It was concluded that each of the “enhanced” vaccines induced cellular and humoural responses superior to standard formulations.

It should be noted that UV-inactivated virus was used in this comparison, which can only stimulate CD8+ T cells through antigen cross-presentation to load MHC class 1 molecules. There are essentially no HA epitopes in humans that stimulate CD8+ T cells (in contrast to mouse models where there are many). Thus, responses will be mostly dependent on helper T cells, emphasizing the potential importance of the “enhanced” formulations in stimulating polyfunctional CD4+ responses. These results contrast with earlier data from Sridhar et al. [4] and Wilkinson et al. [5] which are repeatedly referenced as correlates of protection, but which were generated based on data from a model of direct loading of MHC class 1 molecules with peptides representing viral nucleoprotein (NP) and matrix (M) protein. The Li et al. [3] paper is therefore important because it justifies the use of the only enhanced vaccine that contains these internal proteins. Most important for future work will be detailed studies of CD4+ and CD8+ T cell responses to virus challenge and related frequencies of cells activated by NP/M peptides, in particular aspects of polyfunctionality assessing the balance between “pro-inflammatory” cytokines (eg. interferon-gamma, tumour necrosis factor, IL 2) and “anti-inflammatory” cytokines (eg. IL 4, IL 10) which can be very informative as “correlates of protection” in other circumstances [6].

## Data Availability

not applicable.
